# Environmental Exposure of Arsenic in Groundwater Associated to Carcinogenic Risk in Underweight Children Exposed to Fluorides

**DOI:** 10.3390/ijerph17030724

**Published:** 2020-01-22

**Authors:** Nelly Molina-Frechero, Martina Nevarez-Rascón, Omar Tremillo-Maldonado, Marcela Vergara-Onofre, Rey Gutiérrez-Tolentino, Enrique Gaona, Enrique Castañeda, Lizet Jarquin-Yañez, Ronell Bologna-Molina

**Affiliations:** 1 Division of Biological and Health Sciences, Universidad Autónoma Metropolitana, Mexico City 04460, Mexico; nmolinaf@hotmail.com (N.M.-F.); mvergara@correo.xoc.uam.mx (M.V.-O.); reygut@correo.xoc.uam.mx (R.G.-T.); gaen1310@correo.xoc.uam.mx (E.G.); rcastane@correo.xoc.uam.mx (E.C.); 2 Faculty of Dentistry, Universidad Autónoma de Chihuahua, Chihuahua 31000, Mexico; martina.nevarez@gmail.com; 3 Research Department, Faculty of Dentistry, Universidad Juárez del Estado de Durango, Durango 34000, Mexico; oatm88@msn.com; 4 Center of Applied Research in Environment and Health, CIACYT, Universidad Autónoma de San Luis Potosí, San Luis Potosí 78120, Mexico; draljarquin@hotmail.com; 5 Molecular Pathology, School of Dentistry, Universidad de la República, Montevideo 11600, Uruguay

**Keywords:** arsenic, exposure dose, hazard quotient, non-carcinogenic, carcinogenic risk

## Abstract

Background: The purpose of this study was to determine the concentration of inorganic arsenic (As) in the potable water available to the population to be able to estimate the non-carcinogenic risks for underweight children and the carcinogenic risk for adults exposed to As intake who live in the Mezquital municipality, Durango, Mexico. Methods: The As content was quantifed in the water supply sources for human use and its intake was estimated in Mezquital population, southern Durango. With the data obtained, the hazard quotient (HQ) was calculated to determine the non-carcinogenic risk to develop chronic systemic effects in underweight children. The Environmental Protection Agency (EPA) reference health values estimating As exposure risk are from 0.0003 mg/kg/day (non-carcinogenic) to 1.5 mg/kg/day (carcinogenic risk). Results: The analyzed waters presented as concentrations that varied from 0.3 to 10.2 µg/L, with a mean of 7.35 µg/L (CI 95% 6.27–8.38). The exposure dose was 0.4 to 1.36, and the HQ was 1.90 to 6.48 mg/kg/day, the estimated carcinogenic risk from adults varied from 1.28 to 4.37E^−4^, with values of 3.74–4.37E^−4^ mg/kg/day in central area. Conclusions: The children are at risk to develop chronic systemic effects due to ingestion of As from water.

## 1. Introduction

Arsenic (As) that is found in water at high concentrations poses a health risk when ingested. The most toxic variable of inorganic As is found in geothermal water sources and groundwater [1,2].

This element is identified in drinking water, which is a health problem that is spread worldwide. 4% of the population in Mexico is exposed to As in elevated concentrations [3]; meanwhile, some authors report As exposure in various states near the center and north of the country [4,5]. The state of Durango has high concentrations of fluoride in water, with a high incidence of dental fluorosis [5,6]. Therefore, in addition to as, these areas are overexposed to fluorides, and studies have reported synergy of fluorides and arsenic, with monitoring by water control organisms [7]. In Mexico there are areas exposed to as and fluoride.

As has been classified at the international level as a potent carcinogen by the International Agency for Research on Cancer [8]. Due its association to other side effects on the cardiovascular and nervous system, As concentrations are widely recognized as a priority health issue [9,10].

The WHO reports that arsenicosis can be induced by chronic exposure above 0.5 mg/day [11,12]. Lower intelligence quotient, as a neurological effect, and impaired immune response have been associated with exposure to children [13]. Thus, in order to avoid these health problems in the population exposed and managing adequately the resources that are destinated to solve this situation, new guides must be established with a content focused on the evaluation of risks. The aim of this study was to determine the concentration of inorganic As in the potable water available to the population to be able to estimate the non-carcinogenic risks for underweight children and the carcinogenic risk for adults exposed to As intake who live in the Mezquital municipality, Durango, Mexico.

## 2. Materials and Methods

### 2.1. Ethical Considerations

This study was reviewed and approved by the Ethics Committee at the Autonomous Metropolitan University-Xochimilco DCBS#3450491-CE.2018.009 and by the Ethics Committee at the Juarez University of Durango State EC-FO-UJED-01-14.

### 2.2. Study Sites

**Figure 1:**
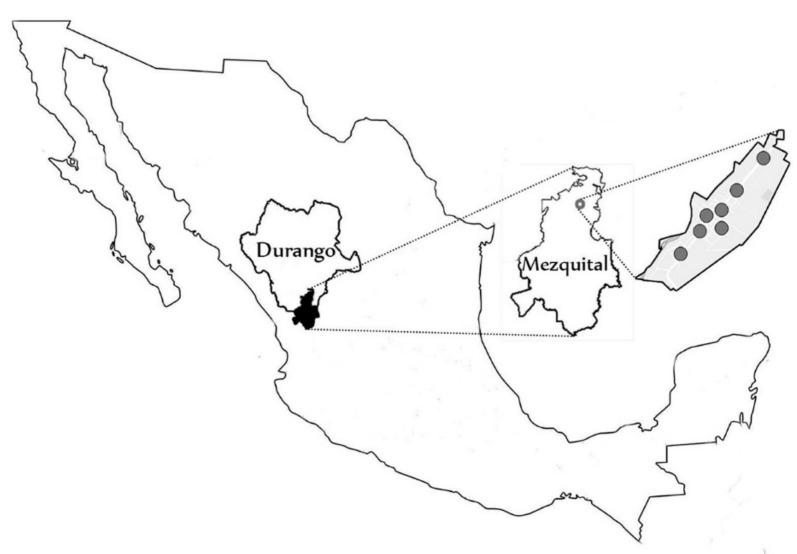
Map depicting the Mezquital south of the state of Durango in Mexican Republic.

Mezquital municipality is located in the extreme south of the state of Durango (Figure 1), in a mountainous area, at an altitude of 2053 meters above sea level. The mean annual temperature fluctuates between 20 and 24 °C, reaching 40 °C in warm seasons and during sunny hours. This area is predominantly inhabited by indigenous groups. It consists of a population of approximately 3528 children between 1 and 3 years of age, with underweight, according to data from the INEGI-SEP [14].

### 2.3. Determination of Fluoride

Water samples were collected in polyethylene bottles washed with deionized water; those that were labeled with the sample number, the well and the location for identification. Fluoride analysis was performed with the potentiometric method and ion selective electrode. A 4-star fluoride ion selective Orion electrode from Thermo Electron Corporation was used. A calibration curve was prepared using standard solutions with concentrations between 0.01 and 10 mg/L. TISAB III was added to the standards to stabilize the ionic strength [15]. Fluoride readings for each sample were recorded using the Star Navigator and LabSpeed Navigator software packages.

### 2.4. Determination of Arsenic

#### Water Samples

Twenty-one water samples were taken from the urban zone of Mezquital; the samples were from the south, center and north areas of the municipality, and the collected water was intended to be used for human consumption and agricultural irrigation [16].

Water was collected in sterile one-liter amber colored glass containers with screw caps to determine the concentration of heavy metals. The procedure described in international norm ISO 11047(1998), was applied for heavy metal determination by flame atomic absorption spectrometry (AAS), for which a Varian double-beam spectrophotometer, model Spectrum AA220, with an air/acetylene flame and nitrous oxide/acetylene and a VGA 77 hydride generator was used, following the method of Jiménez et al. 1996; for quality control, a standard reference with specific concentrations of As was used. Once the apparatus was calibrated, the samples were analyzed in triplicate. In the case that the duplicate analyses disagreed, both were discarded, and the measurement was repeated [17,18].

### 2.5. Estimation of Exposure Dose

The daily exposure dose (DED), expressed as mg/kg/day, was estimated based on the results obtained from the As quantification (mg/L), the water daily ingestion rate (L/day) divided by the body mass of the individual (kg):

$$\mathrm{DED}=\frac{\left(\mathrm{As}\;\mathrm{Concentration}\;\mathrm{mg}/L\right)\;\left(\mathrm{Water}\;\mathrm{intake}\;L/d\right)}{\left(\mathrm{Body}\;\mathrm{mass}\;\mathrm{kg}\right)}$$

To calculate the exposure dose, the data obtained from the concentration of As in water were used, taking into account for this estimation water intake values according to the Institute of Public Health [19] and the National Institute of Pediatrics [20] for children aged 12 months to three years and considering that the children may drink increased amounts of liquids because of the weather, which can reach 40 °C. The weight of the children was considered to be 10.5 kg, given that in that study area, the children are of underweight according to INEGI data [14]. The estimations were based on the following information: a child consumes a daily a quantity of water in liters that can come from different sources.

### 2.6. Risk Assessment

Subsequently, the risk or hazard quotient (HQ) was determined according to the following formula [21]:

$$\mathrm{HQ}=\frac{\left(\mathrm{EF}\right)\left(\mathrm{ED}\right)\left(\mathrm{WI}\right)\left(\mathrm{AsC}\right)}{\left(\mathrm{RfD}\right)\;\left(\mathrm{BW}\right)\left(\mathrm{AT}\right)}$$

EF is the Exposure Frequency (from 365 days/year), ED is the Exposure Duration (for adults 70 years, children 12 month-three years), WI is the Water Intake (L/d), AsC is the As Concentration (mg/L), RfD is the Oral Reference Dose (mg/kg/day), BW is the Body Weight, AT is the Average exposure Time (equal to EFxED).

If the HQ dose is greater than one (HQ > 1), the exposed population is at risk of developing chronic systemic effects.

The oral reference dose (RfD) corresponds to an estimate of daily exposure that avoids bodily harm and can be calculated for both carcinogenic and noncarcinogenic substances.

Table 1 shows the reference values according to the EPA. This table indicates that for an As intake between the no observed adverse effect level NOAEL (intake limit associated with the absence of clinical manifestations of adverse effects in exposed individuals) and the lowest observed adverse effect level LOAEL (limit of Ingestion associated with the clinical manifestation of low magnitude adverse effects), exposed subjects may present hyperpigmentation, hyperkeratosis or possible vascular complications.

If the exposure exceeds the LOAEL, the subject is at risk of developing cancer in internal organs and other types of more severe injuries [22].

### 2.7. Estimation of Carcinogenic Risk

For carcinogenic risk, exposure to As was estimated for the adult population considering that carcinogenic risk (CR), in general, increases throughout life; therefore, the reference was determined for an individual who consumes 2 L of water/day and weighs 70 kg [22]. This is based on the EPA [9] and ATSDR [22,23], and therefore, it is calculated considering the exposure dose by the cancer dependent factor (CSF), which is 1.5 (Table 1). To verify the validation of the calculated data, the following formula was applied:

$$\mathrm{CR}=\frac{\left(\mathrm{EF}\right)\left(\mathrm{ED}\right)\left(\mathrm{WI}\right)\left(\mathrm{AsC}\right)\left(\mathrm{CSF}\right)}{\left(\mathrm{BW}\right)\left(\mathrm{AT}\right)}$$

### 2.8. Statistical Analysis

The data obtained for triplicate measurements data were analyzed using univariate statistical methods, and means, standard deviations and 95% confidence intervals for each sample were calculated; continuous variables were analyzed by nonparametric tests Kruskal–Wallis, *p*-value <0.05 was considered statistically significant. The IBM SPSS version 21 (Armonk, NY, USA) software package was used for data analysis.

## 3. Results

Twenty-one water samples were analyzed from the seven areas of the Mezquital municipality, the fluoride concentration were between 7.87 and 9.23 mg/L, mean 8.414 ± 0.155. The area of the central region the most populated where the greatest number of children reside, mean water fluoride was 8.136 (CI 95% 7.941–8.332). The least populated north and south region averaged 9.068 (CI 95% 8.901–9.235), and 8.217 (CI 95% 8.179–8.255) respectively. These results are presented in Table 2 and Table 3.

Table 4 shown the concentrations of arsenic in water found the mean was 7.351 (CI 95% 6.272–8.381) and ranged from 3 to 10.2 (mg/L^−3^).

**Figure 2:**
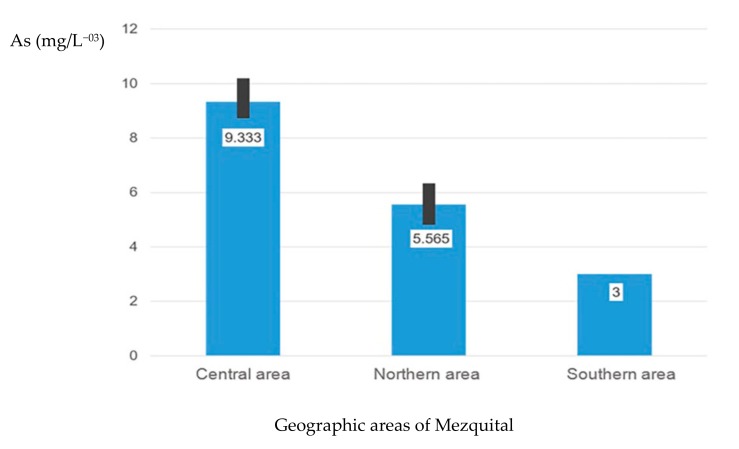
Mean As concentrations in Mezquital Durango in the three geographic regions.

Figure 2 shows the mean of As in the three geographic regions, the central area with mean 9.33 (CI 8.344–10.323) is the zone with higher As levels. The differences of mean of As in water per area (*p* = 0.002).

Table 5 shows the estimation of the daily exposure doses (DED mg/kg/day), and the non-carcinogenic risk (HQ unitless) for children. It can be observed that children are at risk to develop chronic systemic effects (HQ > 1) due to the daily intake of As from water, which is higher than those recommended: RfD = 0.0003 (mg/kg/day).

Table 6 shows the estimation of the daily exposure dose (DED mg/kg/day), the non-carcinogenic risk (HQ) and the carcinogenic risks (CR) for adults. It can be observed that the daily intake of As is not higher than those recommended, thus, the adults are not at risk to develop chronic systemic effects (HQ < 1) due to the ingestion of As from water, but the CR is above the acceptable lifetime risk. According to the EPA (2000), if CR results above of Acceptable Lifetime Risk (ALR), equal to 1 × 10^−5^, there is 1 in 100,000 chance that a person may develop cancer from the oral exposure to inorganic As. CR obtained in this study for the adult population is a greater health concern because, in the order of 1 × 10^−4^ (1.29 × 10^−4^ < CR < 4.37 × 10^−4^), there is around 1 chance over 10,000 that a person may develop cancer.

## 4. Discussion

The water of the Mezquital municipality has high concentrations of fluoride that are similar to the highest in the Durango city. Thus, the child population is the most susceptible to overexposure to fluorides. There are also other risk factors such as calcium deficiency, malnutrition, and kidney disorders that affect the acid-basic balance. All these factors may be causing a greater impact on the children population exposed to as and fluoride. The presence of this concentration of fluoride may have a synergistic effect with the As [24].

As is classified as a toxic chemical and carcinogenic element, representing a serious environmental problem in Mexico and the world. When there is a greater natural geological presence of As, high levels can be found in groundwater and geothermal water [25,26]. In the present study, 14.28% of the samples exceeded WHO and EPA limits. The DED was 0.0006 to 0.0019 mg/kg/day, and the HQ estimation that children presented was 1.90 to 6.48, indicating that children in these populations are at risk of developing chronic systemic effects due to ingestion of As through water.

As indicated by our results, these children are at risk of keratosis, hyperpigmentation, and possible vascular complications. In addition, the effects of As exposure range from acute lethality to chronic effects, with multiple consequences, in which several different systems and organs, including the skin and respiratory tract, cardiovascular, immune, reproductive and genitourinary and nervous systems, and endocrine, erythropoietic, hepatic and renal systems can be affected [27]. Most health effects are related to chronic As exposure, which affects almost all organs and systems of the body, the most common being chronic hydroarsenicism. Even in some countries, black foot disease has been reported, causing gangrene in the feet [27,28].

As is recognized as a carcinogenic substance and registered in the IARC. As exposure has genetic and epigenetic effects through a series of reactions at the cellular level; therefore, inorganic compounds of arsenic are clearly carcinogenic [29]. The most well-known effects are on lung tissue, which occur with high As exposure.

In addition, it can be observed that the daily intake of As is not higher than recommended and the HQ of adults is less than one (HQ < 1), which may indicate that this population is not at risk of developing chronic systemic effects. Otherwise, the estimation of the CR varied from 1.29 to 4.37 E^−4^, with values that ranged from 3.74 to 4.37E^−4^ mg/kg/day in central area, indicating that if these children continue to be chronically exposed to As throughout life to adulthood, may have a higher risk, above the ALR, of developing cancer.

In this study, the exposure doses in the southern and northern areas of the community were lower than the safety doses; the population in the center of the community, with the largest number of houses and schools, had higher exposure doses than what is safe. It has been reported in other states, such as Hidalgo and Zacatecas, that diverse health effects are related to As in water. These estimates suggest that a high percentage of children can be affected, when adults, by multiple health problems [29,30,31].

This population, in addition to being exposed to high concentrations of fluoride in water, suffers from multiple nutritional deficiencies, and this population is underweight and, due to high temperatures, ingests many fluids, placing them at an increased risk, which may be underestimated due the food grown in this areas may have high As concentration and also may be a potential source of organic As; this could be taken as an important topic for further studies in the same area with the assessed population of the current study.

Populations with nutritional deficiencies, such as in vitamins C and A and methionine, are associated with greater toxic effects because the defense mechanism of the body may be insufficient, e.g., in the case of arsenic. A diet with low consume of folate decreases As metabolism, which can increase the possibility of the population to be affected. Therefore, interventions to reduce environmental exposure are justified [29]. In terms of costs, in populations in India, China and Bangladesh, greater severity has been reported for As exposure than for diseases such as cancer, as these are populations with nutritional deficiencies [32]. Other studies in populations in the United States where As concentrations in drinking water are the same present no evidence of arsenism; it is likely that these populations are well nourished and have a high socioeconomic status and, thus, a higher level of protection, unlike populations living in developing countries [33].

## 5. Conclusions

Although some of the original water samples were below the permissible limits, considering the characteristics of the studied area in the rainy season and that the region is exposed to high concentrations of fluoride in water, in addition to nutritional deficiencies and a low socioeconomic status, we conclude that the population is exposed to a significant risk due to As exposure and that it is essential to disclose these circumstances so that the relevant agencies consider the situation as a serious public health problem.

The intake of pollutants in water must be regulated, which results in a priority task to be accomplished; without performing interventions and dissemination actions, such pollutants will have health effects and can even cause cancer.

In designing an intervention, childhood malnutrition and the sensitivity of children to increased risks of adverse effects caused by an intake of high concentrations of As in water should be kept in mind when quality drinking water be provided to the communities (e.g., installing treatment plants), either communicating the risks through health care campaigns, and finally regarding the risks of drinking contaminated water, keeping the epidemiological surveillance.

Providing new information based on risks and health improvement destinated to the population that reside in As contaminated areas is the most important contribution of the present study, which results in taking better decisions in order to prevent adverse effects in people that are constantly exposed.

We, therefore, conclude that the underweight children of the studied population are at risk to develop chronic systemic effects and will present in the future major CR. Thus, exposure to As mainly through water, which is a public health problem that has become relevant in recent years due to the development of research on this heavy metal that, along with other elements such as fluoride, may have a toxic synergistic effect on humans.

## Figures and Tables

**Table 1:** Reference health values for estimating As exposure risk.

Estimated Health Risk	Reference Value	Definition	Value	Unit	Critical Effects	Organization
Noncarcinogenic	NOAEL	No observed adverse effect level	0.0008	mg/kg/day	Hyperpigmentation, keratosis and vascular complications	EPA
Noncarcinogenic	LOAEL	Lowest observed adverse effect level	0.014	mg/kg/day	Hyperpigmentation, keratosis and vascular complications	EPA
Noncarcinogenic	RfD	Reference dose	0.0003	mg/kg/day	Hyperpigmentation, keratosis and vascular complications	EPA
Carcinogen	CSF	Cancer-dependent factor	1.5	mg/kg/day	Cancer	EPA

EPA—United States Environmental Protection Agency.

**Table 2:** Fluoride concentration by region of the Mezquital municipality in (mg/L).

Geographic Region	*n*	Minimum	Maxima	Mean	±SD
C1	3	8.63	8.65	8.637	0.0115
C2	3	7.87	7.88	7.877	0.0058
C3	3	8.04	8.06	8.048	0.0115
C4	3	7.98	7.99	7.987	0.0058
N1	3	8.92	8.93	8.923	0.0058
N2	3	9.20	9.23	9.213	0.0153
S1	3	8.20	8.23	8.217	0.0153
Total	21	7.87	9.23	8.414	0.1553

C—central area; N—northern area; S—southern area; ±SD—standard deviation.

**Table 3:** Mean fluoride concentration of the Mezquital municipality in (mg/L).

Geographic Region	*n*	Minimum	Maxima	Mean	CI 95%
Central	12	7.87	8.65	8.14	7.94–8.33
Northern	6	8.92	9.23	9.07	8.90–9.23
Southern	3	8.20	8.23	8.217	8.178–8.255
Total	21	7.87	9.23	8.414	8.192–8.637

CI—confidence interval.

**Table 4:** As (mg/L^−3^) in drinking water in Mezquital.

Geographic Region	*n*	Range	Minimum	Maxima	Mean (mg/L^−3^)	±SD	CI 95%
C1	3	0.180	9.140	9.320	9.200	0.103	9.140–9.320
C2	3	0.080	10.160	10.240	10.200	0.014	10.160–10.240
C3	3	0.100	9.170	9.270	9.213	0.192	9.170–9.270
C4	3	0.050	8.690	8.740	8.720	0.016	8.690–8.740
N1	3	0.040	6.300	6.340	6.320	0.007	6.300–6.340
N2	3	0.070	4.780	4.850	4.810	0.013	4.780–4.850
S1	3	0.040	2.800	3.200	3.000	0.071	2.800–3.200
	21	0.140	2.800	10.240	7.351	0.278	6.272–8.381

C—central area; N—northern area; S—southern area; ±SD—standard deviation; CI—confidence interval.

**Table 5:** As exposure dose and estimation of non-carcinogenic risk in the underweight child population.

Geographic Region	*n*	As (mg/L)	DED (mg/kg/day)	HQ
C1	3	0.00920	0.0018	5.84
C2	3	0.01020	0.0019	6.48
C3	3	0.00921	0.0018	5.85
C4	3	0.00872	0.0017	5.54
N1	3	0.00632	0.0012	4.01
N2	3	0.00481	0.0009	3.05
S1	3	0.00300	0.0006	1.90

C—central area; N—northern area; S—southern area; DED—daily exposure dose; HQ—non-carcinogenic risk.

**Table 6:** As in water, exposure dose and carcinogenic risk estimation in the adult population.

Geographic Region	*n*	As (mg/L)	DED (mg/kg/day)	HQ	CR
C1	3	0.00920	0.0003	0.876	3.94E^−4^
C2	3	0.01020	0.0003	0.971	4.37E^−4^
C3	3	0.00921	0.0003	0.877	3.94E^−4^
C4	3	0.00872	0.0002	0.830	3.73E^−4^
N1	3	0.00632	0.0002	0.601	2.71E^−4^
N2	3	0.00481	0.0001	0.458	2.06E^−4^
S1	3	0.00300	0.0001	0.285	1.29E^−4^

C—central area; N—northern area; S—southern area; DED—daily exposure dose; CR—carcinogenic risk.
